# Supplementary material for: Multi‐dataset identification of innovative feature genes and molecular mechanisms in keratoconus
Source: J Cell Mol Med. 2024 Sep 19;28(18):e70079. doi: 10.1111/jcmm.70079 (PMC11412914; doi:10.1111/jcmm.70079)
Supplement: Supplementary file 2 — Material S2. [file JCMM-28-e70079-s002.zip › 7.GSEA analysis/1.ARL11/KEGG_STEROID_HORMONE_BIOSYNTHESIS.html]

Details for gene set KEGG\_STEROID\_HORMONE\_BIOSYNTHESIS[GSEA]

|  || Dataset | ARL11.group.cls#h\_versus\_l |
| Phenotype | group.cls#h\_versus\_l |
| Upregulated in class | l |
| GeneSet | KEGG\_STEROID\_HORMONE\_BIOSYNTHESIS |
| Enrichment Score (ES) | -0.3057273 |
| Normalized Enrichment Score (NES) | -0.94462806 |
| Nominal p-value | 0.56633663 |
| FDR q-value | 1.0 |
| FWER p-Value | 1.0 |
Table: GSEA Results Summary

  

Fig 1: Enrichment plot: KEGG\_STEROID\_HORMONE\_BIOSYNTHESIS      
 Profile of the Running ES Score & Positions of GeneSet Members on the Rank Ordered List

  

| SYMBOL | TITLE | RANK IN GENE LIST | RANK METRIC SCORE | RUNNING ES | CORE ENRICHMENT || 1 | UGT2B7 | na | 1178 | 0.368 | 0.0119 | No |
| 2 | CYP7A1 | na | 2190 | 0.283 | 0.0181 | No |
| 3 | CYP11A1 | na | 2342 | 0.274 | 0.0479 | No |
| 4 | UGT2B15 | na | 2914 | 0.244 | 0.0620 | No |
| 5 | UGT2B11 | na | 2923 | 0.244 | 0.0922 | No |
| 6 | SRD5A2 | na | 3151 | 0.234 | 0.1148 | No |
| 7 | CYP3A7 | na | 3477 | 0.220 | 0.1329 | No |
| 8 | CYP21A2 | na | 3560 | 0.217 | 0.1576 | No |
| 9 | UGT2B4 | na | 3576 | 0.216 | 0.1842 | No |
| 10 | UGT1A8 | na | 3762 | 0.209 | 0.2050 | No |
| 11 | STS | na | 3978 | 0.201 | 0.2239 | No |
| 12 | HSD3B1 | na | 4228 | 0.192 | 0.2407 | No |
| 13 | UGT1A6 | na | 4803 | 0.172 | 0.2456 | No |
| 14 | UGT2B28 | na | 5683 | 0.146 | 0.2385 | No |
| 15 | HSD17B3 | na | 7093 | 0.110 | 0.2116 | No |
| 16 | CYP3A5 | na | 7636 | 0.099 | 0.2083 | No |
| 17 | HSD17B12 | na | 7822 | 0.096 | 0.2149 | No |
| 18 | UGT2B10 | na | 9893 | 0.062 | 0.1629 | No |
| 19 | SRD5A1 | na | 10582 | 0.053 | 0.1497 | No |
| 20 | UGT2A3 | na | 13634 | 0.022 | 0.0644 | No |
| 21 | CYP3A4 | na | 14527 | 0.015 | 0.0405 | No |
| 22 | HSD17B1 | na | 15637 | 0.006 | 0.0094 | No |
| 23 | COMT | na | 16431 | 0.001 | -0.0134 | No |
| 24 | HSD3B2 | na | 17798 | -0.009 | -0.0517 | No |
| 25 | HSD17B8 | na | 17839 | -0.009 | -0.0517 | No |
| 26 | SULT2B1 | na | 18139 | -0.011 | -0.0589 | No |
| 27 | CYP17A1 | na | 18931 | -0.016 | -0.0797 | No |
| 28 | HSD17B7 | na | 19955 | -0.023 | -0.1062 | No |
| 29 | CYP11B1 | na | 20068 | -0.024 | -0.1064 | No |
| 30 | UGT2A1 | na | 20689 | -0.029 | -0.1207 | No |
| 31 | HSD11B2 | na | 23419 | -0.055 | -0.1926 | No |
| 32 | SRD5A3 | na | 27257 | -0.114 | -0.2890 | No |
| 33 | CYP11B2 | na | 27838 | -0.127 | -0.2899 | Yes |
| 34 | CYP7B1 | na | 28122 | -0.134 | -0.2813 | Yes |
| 35 | AKR1C4 | na | 28839 | -0.153 | -0.2829 | Yes |
| 36 | HSD17B2 | na | 29040 | -0.158 | -0.2690 | Yes |
| 37 | AKR1C1 | na | 29392 | -0.169 | -0.2580 | Yes |
| 38 | AKR1D1 | na | 29648 | -0.177 | -0.2433 | Yes |
| 39 | AKR1C3 | na | 30128 | -0.191 | -0.2332 | Yes |
| 40 | HSD17B6 | na | 31252 | -0.230 | -0.2369 | Yes |
| 41 | CYP3A43 | na | 32273 | -0.273 | -0.2322 | Yes |
| 42 | HSD11B1 | na | 33227 | -0.339 | -0.2174 | Yes |
| 43 | CYP19A1 | na | 33509 | -0.360 | -0.1805 | Yes |
| 44 | CYP1A1 | na | 34339 | -0.484 | -0.1440 | Yes |
| 45 | SULT1E1 | na | 34396 | -0.498 | -0.0835 | Yes |
| 46 | CYP1B1 | na | 34694 | -0.745 | 0.0010 | Yes |
Table: GSEA details [plain text format]

  

Fig 2: KEGG\_STEROID\_HORMONE\_BIOSYNTHESIS      
 Blue-Pink O' Gram in the Space of the Analyzed GeneSet

  

Fig 3: KEGG\_STEROID\_HORMONE\_BIOSYNTHESIS: Random ES distribution      
 Gene set null distribution of ES for **KEGG\_STEROID\_HORMONE\_BIOSYNTHESIS**
